# Exploring usage pattern variation of free-floating bike-sharing from a night travel perspective

**DOI:** 10.1038/s41598-024-66564-2

**Published:** 2024-07-11

**Authors:** Senbin Yu, Xianke Han, Ling Liu, Gehui Liu, Minghui Cheng, Yu Ke, Lili Li

**Affiliations:** 1 Key Laboratory of Urban Rail Transit Intelligent Operation and Maintenance Technology & Equipment of Zhejiang Province, Zhejiang Normal University, Jinhua, 321004 China; 2 College of Engineering, Zhejiang Normal University, Jinhua, 321004 China; 3 Transport Planning and Research Institute Ministry of Transport, Beijing, 100028 China; 4 Beijing National Railway Research & Design Institute of Signal &, Communication Group Co., Ltd., Beijing, 100070 People’s Republic of China; 5 School of Transportation and Logistics, Southwest Jiaotong University, Chengdu, 611756 China; 6 School of Transportation Engineering, East China Jiaotong University, Nanchang, 330013 China; 7 Jinhua Rail Transit Operation Co. Ltd, Jinhua, 321004 China

**Keywords:** Free-floating bike sharing, Usage pattern, Night travel, Built-environmental effect, Temporal-spatial distribution, Environmental impact, Socioeconomic scenarios, Sustainability, Civil engineering, Statistics

## Abstract

Free-floating bike sharing (FFBS) attracts increasing research focusing on usage patterns, determining factors, and integrated transportation. However, existing researchers tend to overlook the variation in usage characteristics over various time ranges, particularly the usage pattern at night. This paper is conducted to fill the gap through a series of analysis approaches on FFSB in Beijing. The characteristics of the usage pattern, including time-varying usage and traveling distance distributions, are initially illustrated. Subsequently, the spatial patterns of FFBS are visualized and thoroughly analyzed in different time ranges and origin-destination (O-D) flows. A statistical model evaluating the environmental effects of FFBS trips revealed the source of FFBS usage. In addition to focusing on the nighttime, the usage patterns varying day and night are compared through the analysis. The findings explain the usage pattern variation and the unique pattern at night, providing valuable insight for improving the management of the FFBS system.

## Introduction

### Background

Bike-sharing systems (BSS) have grown swiftly in cities since the 2000s. Until January 2021, survey^1^ reports 2007 bike-sharing programs and approximately 9,440,776 bikes in service worldwide. Compared to motor-based travel, cycling activities are considered economical, flexible, and novel methods to mitigate traffic congestion^2^. Free-floating bike sharing (FFBS) systems such as Mobike gradually showed up with modern technology development. Compared to the dock-based BSS (DBSS), the FFBS system (FFBSS) allows users to rent a bike and return it almost anywhere as long as they adhere to traffic rules^3^. The short walking distance for reaching a bike from FFBSS promotes its development with increasing popularity and unprecedented speed in many large cities worldwide.

FFBS is equipped with locator devices that generate itinerary records, including renting time, returning time, and real-time position trajectory. These digital footprints benefit in portraying user behaviors, predicting travel demands, and improving city-wide traffic systems. Accordingly, researchers have gradually focused on BSS users' characteristics and behaviors from theory and practice perspectives^4^. However, due to the privacy issues for users and the data security of operator companies, understanding FFBS usage and related influence factors is still insufficient because of the difficulty of data availability^5^.

The characteristics of FFBS usage observably vary within a day, resulting in a heterogeneous usage pattern^6^. The well-explained regular patterns in the daytime, especially at peak hours, provide beneficial instructions for FFBS rebalancing management^7^ and integrated transportation operation^8^. Understanding the differences in time-varying usage patterns through exploring irregular patterns in overlooked time periods (such as the nighttime) is crucial for FFBS usage explanation and urban travel service improvement.

### Usage pattern of free-floating bike sharing

Existing studies on FFBS are mainly grouped into two streams. The first stream is the fundamental analysis of basic statistics property of FFBS trips (e.g., travel time, travel distance) and the built-environmental effects (e.g., travel purpose). The built environment, primarily comprised of various Points of Interest (POI), is one of the key factors contributing to the generation of bike-sharing demand. Shen et al.^9^ investigated the impact of fleet size, built environment, and weather conditions on the usage of FFBS by a spatial autoregressive model. Tu et al.^10^ proposed a generalized additional mixed model to distinguish built-environmental factors affecting FFBS usage density. Several factors positively associated with the density of FFBS usage were recognized, such as the floor area ratio, residential, green space, et al. A model framework was proposed by Du et al.^11^ for investigation in various temporal-spatial scales in Shanghai, China. They found that the lognormal distributions can fit the travel distance and travel time of FFSB trips. The usage of FFBS is mainly concentrated in fewer hotspots surrounding metro stations. Li et al.^12^ explored the characteristics of FFBS usage based on questionnaires, and they found that short-distance trips were mainly used primarily for commuting during rush hours in urban areas. Du and Cheng^13^ also presented similar usage patterns. Ji et al.^14^ addressed the effects of street structure and land use on bike sharing, which is fitted as an inverted U-shape association. Chen and Wei^15^ explored the impacts of the built environment on bike sharing on weekends. Recreational and residential areas have been proven to impact bike-sharing usage positively.

The differences in usage characteristics between DBSS and FFBSS have been found in previous studies. Ji et al.^2^ examined their usage regularity and determinants, and different spatial–temporal distribution was observed. The results showed that the trip distance of DBSS is longer than that of FFBSS, which is consistent with the previous research^16^. The distribution of production and attraction of bike-sharing trips (i.e., the imbalanced demand) existed across different periods^17^. FFBSS generates a self-organized bike flow and spatial features throughout the city. Recent research^18^ focused on the spatial characteristics of the imbalance of bikes due to one-way trips. It revealed a power-law distribution in the unbalanced nodes by constructing an FFBS mobility origin-destination network. Besides, Xing et al.^4^ investigated the travel patterns and trip purposes related to point-of-interests (POI) and found that dining and bike-and-ride are the top two purposes.

The second stream integrates BSS and urban transportation systems, namely, the bike-and-ride^8,19^. BSS serves as a time-saving solution for alleviating the first/last mile problem, an inherent defect of an urban transportation system. A case in Guangzhou, China, reveals that 63% of bike-sharing trips connect with urban transportation systems^20^. Zhou et al.^21^ also reported that 35.3% of respondents intended to choose FFBS as an effective mode for accessing metro stations after the operation of FFBSS in Shanghai. According to a case study with a practical distinguishing approach, Liu et al.^22^ revealed that more than 85% of bike-sharing trips belong to bike-of-ride. Compared with walking and buses, FFBS is more attractive and acts as a faster feeder mode connecting metro stations^23^. The turning from walking to bike sharing as a feeder mode is more pronounced given the extensive usage of FFBS^24^. This integration improves travel efficiency and is one of the crucial reasons FFBS is becoming popular and supports the construction of efficient cities. Also, the metro system's service can be improved by expanding the catchment area using FFSB^24^. Shen et al.^9^ observed the usage patterns of over 10,000 FFBS in Singapore for nine days. They demonstrated that easy access to urban transit systems and supportive facilities promotes the use of FFBSS. Although not dedicated to integrated usage, other research concerning FFBS 5^25^, provides strong evidence of bike-and-ride. Saltykova et al.^26^ indicated that substituting bike sharing for public transit, including car, bus, and subway, may benefit the environment by reducing carbon emissions.

### Usage of bike sharing for night travel

As far as we know, most studies concerning FFBS have focused on regular usage patterns, mainly during the daytime. Few studies have paid close attention to the variation of patterns within a day, embodying the usage heterogeneity^27^. In addition, no publications have revealed the usage characteristics of FFBS at night and the differences among different time ranges. The factors related to usage also remain under-explored. In cities, various mobility patterns exist in different time ranges, requiring different traffic management strategies. Mobility at night, an essential composition of urban travel, is usually overlooked. However, it is now receiving increasing attention due to the growing interest in night economies, transportation policies, and nocturnal activities^28–30^. Night economy describes a wide range of economic activities, mainly service consumption. A thriving night economy means frequent mobility interactions for relaxation and leisure, expecting more public transportation supplies. A well-designed transportation network will promote travel efficiency and travel safety at night. Recently, some research has shed light on the issues of night mobility^31–33^.

Concerning transportation management and planning at night, the urban policy generally lacks an explicit focus on understanding night mobility, including how different people move at night and their needs^28^. There are many reasons for this phenomenon. In practice, related studies have overwhelmingly focused on policy and transportation planning applications during the daytime, especially traffic congestion in peak hours^31^. Although day and night can be seen as the same in human experience, it is argued that existing theory and method structures on night mobility are arranged and oriented toward daytime mobility. The particular features of nocturnal mobility are usually ignored, such as the significance of travel safety, the inconvenience due to the absence of public transportation services, and the cost increment of travel associated with mobility (e.g., the taxi)^28,31^. Urban transit systems like buses or the metro are always absent at night in many cities. As a result, commuters at night have to face more uncertainty, lower public transportation services, and longer travel duration. The availability of night buses is a vital factor influencing people's behavior in China. For example, people prefer to select a taxi rather than a bus to travel a long distance after 18:00^34^. However, taxis are usually expensive for ordinary commuters, who are often not private car users. Their waiting time at stations is extended because of the lower operation frequency of public transportation at night^35^. Besides, due to the concern for personal safety after sunset, commuters at night, especially women, prefer not to wait at outdoor stations^36^, putting a significant burden on low-income commuters^35^.

Apart from the night bus and taxi, the BSS provides another public transportation service at night. The trips of BSSs during the night were usually negligible due to a small proportion of the total usage, especially compared to the daytime^37^. However, the total usage number at night (i.e., from 23:00 to 5:00) had counter-intuitively reached 50,000 a night in Wuhan, China^38^, indicating that BSS usage at night cannot be ignored. This usage pattern of BSS may reveal some of the characteristics of travel at night and improve nighttime transportation management.

Compared with DBSS, FFBSS can seem well-suited for night commutes when public transportation supplies are unavailable^39^. According to a recent survey by Plyushteva^31^, over 10% of tourism and hospitality respondents choose bikes as a primary mode of travel to work at night. They thought bikes offer specific benefits at night: affordability compared with taxis and private cars and more excellent personal safety than walking. Also, due to the difficulties of facility construction and theft prevention, BSS could accommodate the requirement of night commuters rather than private bikes. Also, the benefits of DBSS are still limited by the location of stations for both origin and destination^31^. Users can quickly locate and unlock nearby bikes and park in any proper place due to the absence of fixed docking stations^17^. The advantage of FFBSS is reasonable and is a preferential alternative since the night bus or metro system is bound to miss many areas serviced during the daytime for economic consideration^39^.

### Innovation

Understanding the usage pattern of FBSS at night will help reveal the essential characteristics of night mobility and complement the usage pattern during the day. However, very little research has been conducted into the night travel pattern of FFBS and the relationship between FFBS and the urban transit system, such as night buses. The nighttime provides an excellent observation window to capture the usage pattern and relationship without significant interference, such as with metro services. This relationship is far more complicated as various transportation modes have an advantage over their domain and compete^26^.

In light of the research gaps identified above, this paper represents a novel application of FFBS to analyze the variation of usage patterns in a day. No existing research has systematically and quantitatively discussed spatial–temporal analysis and statistical modeling for using FFBS at night. The generation and mobility requirements at night are crucial elements of this paper and become vital to the pattern variation analysis. A series of studies have been developed to explore an FFBS scheme in Beijing, China. The macroscopic temporal-spatial usage of FFBS at night is first explored given time-varying usage, distribution of travel distance, and layout of usage hotspots. Then, after the four-period division, a statistical method is proposed to illustrate the variety of FFBS usage over a whole day. The differences in the usage patterns arranged by grid cells are found between nighttime and daytime and among four periods. These quantitative comparisons are further visualized and associated with several proposed tools. A multivariable modeling approach is finally applied to explain the generation and attraction of FFBS trips. The relationship between the usage of FFBS and point-of-interests is demonstrated and compared over various periods.

The rest of the paper is organized as follows. The next section visualizes and analyses the usage pattern variation, spatial distributions, and demand sources of FFBS. The discussion, conclusion, and direction for further research are further concluded. The dataset and analysis methods are provided in the last section.

## Results

The nighttime is defined as a time range starting at 23:00 and ending at 5:00 the next day. It is a period that public transportation systems (e.g., metro and day bus) are practically unavailable in Beijing. The remaining time from 5:00 to 23:00 is defined as daytime, including three periods: morning time (5:00–11:00), afternoon time (11:00–17:00), and evening time (17:00–23:00). The weekend nights include Friday night and Saturday night, while the remaining nights of the week belong to the weekday nights. Due to the lack of drop-off time in FFBS records, the FFBS trips are extracted and classified into different periods based on their starting time.

### Time-varying usage and distribution characteristics of travel distance

**Figure 1:**
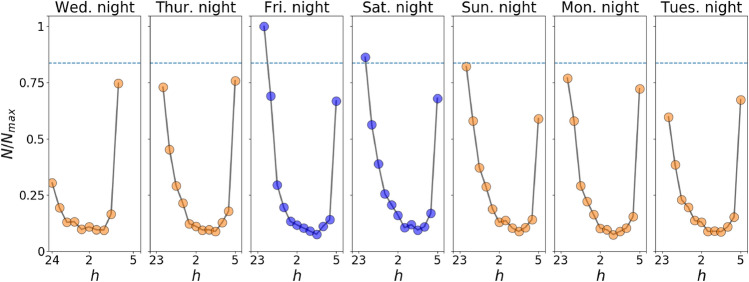
Rescaled time-varying trips per 30 min $N/{N}_{max}$ on nights from 05/10/2017 (Wednesday) to 05/16/2017 (Tuesday), where $N$ is the number of trips and ${N}_{max}$ is the maximum number of trips for all the time individuals. The blue lines indicate the usage variation on weekend nights. It should be noted that the particular time range of the first point on Wednesday is from 0:00 to 0:30, caused by the limitation of the dataset.

To determine quantitative variations of the FFBS usage over nighttime, we investigate the temporal distribution of the number of trips with the 30 min slip ($N$) shown in Fig. 1. Overall, each panel has a common U-shaped tendency of $N$ over the week. Two limited time ranges, 23:00–0:30 and 04:30–05:00, involve approximately 60% of the FFBS usage at night. It reveals that integration with first/last urban transit systems, as one of the primary functions of FFBS^40^, plays a vital role in its usage despite the daily routine and lifestyle. Besides, the number of trips decreases abruptly after 0:00 and reaches the valley floor from 2:30 to 3:30. The reduction in usage is dominated by limited outdoor activity by routine.

In line with other studies (e.g., Böcker et al.^41^), we report on reductions in FFBS usage during the whole days of weekends, as shown in Figure S1, but the usage of FFBS on weekend nights (i.e., Friday and Saturday nights) is slightly larger than that on weekday nights, which is quite the opposite in other periods of a day. This phenomenon is also observed in other studies (e.g., Younes et al.^42^) and can be attributed to vibrant nightlife on weekends^4^. Moreover, trips for recreation, food, and shopping are probably generated with FFBS on weekends when there is no pressing need for travel time^37^. Due to the contrast in usage, the trips for recreation and food can explain why there is more ridership of FFBS on weekend nights, especially the first hour after the service of the public transportation system. The determinants for the promotion of usage will be discussed in the section “Spatial comparison of usage patterns among periods”.

**Figure 2:**
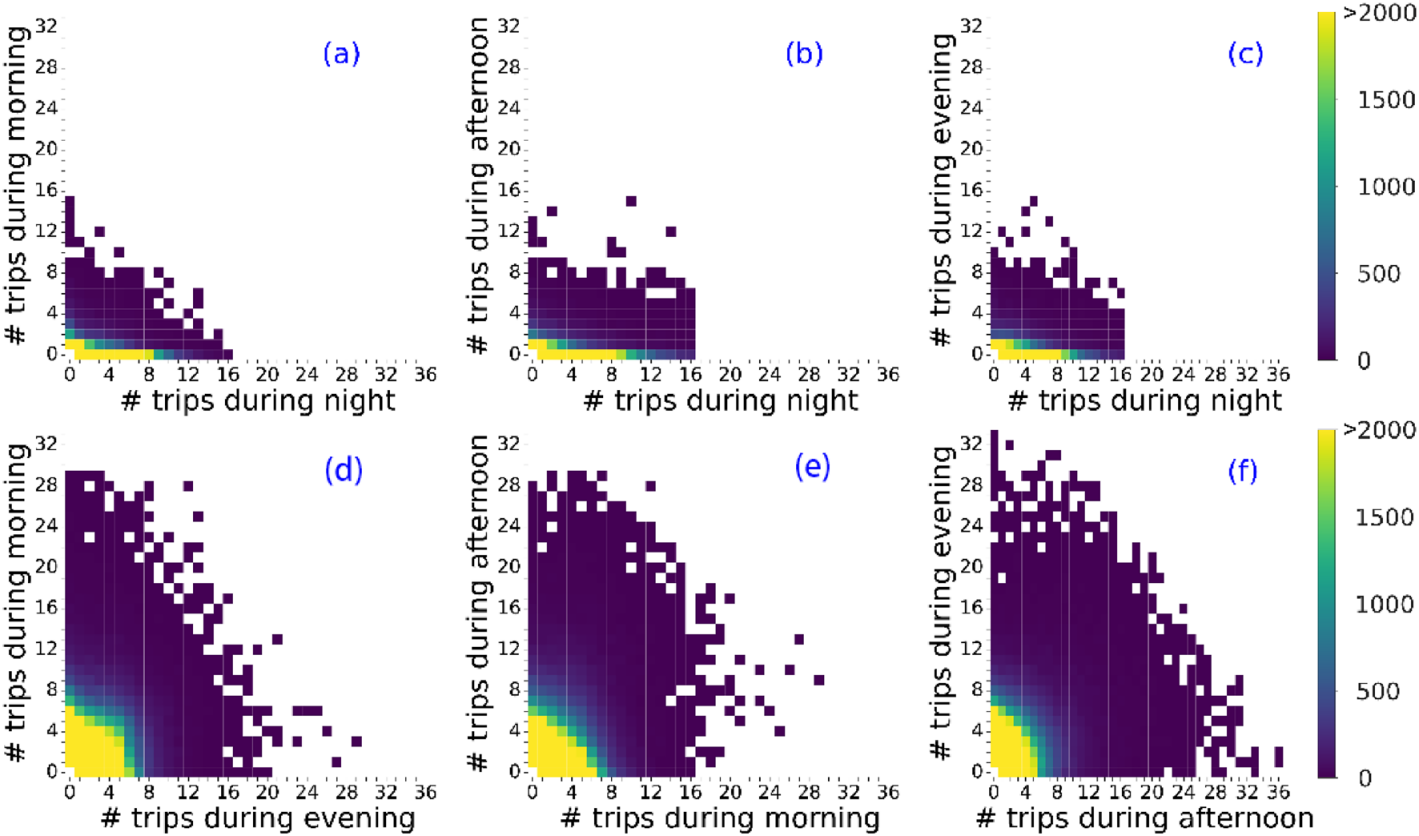
The frequency distribution of FFBS trips during two selected periods: nighttime, morning, afternoon, and evening in Beijing. (**a**) Morning vs. nighttime; (**b**) Afternoon vs. nighttime; (**c**) Evening vs. nighttime. (**d**) Morning vs. evening; (**e**) Afternoon vs. morning, and (**f**) Evening vs. afternoon.

Based on the users' perspective, we further investigate the usage frequency between any two periods, as shown in Fig. 2. The number of trips with the same user is gathered and then categorized into four time ranges. The point with a higher frequency represents more users taking the corresponding scale of trips in both two-time ranges. Interestingly, there is no apparent linear relationship between the usage in nighttime and other periods, consistent with previous results in Beijing^43^. The night users are relatively less than other time users, and they also seldom take bike sharing at other three periods, as shown in Fig. 2a,b, and c. While such a trend is absent in any pair of other periods, some users have few trips in a period but more trips during the other periods, or vice versa. In addition, the usage at night is more random than the other three periods, as evidenced by the higher probability of low-frequency users (see Figure S2).

**Figure 3:**
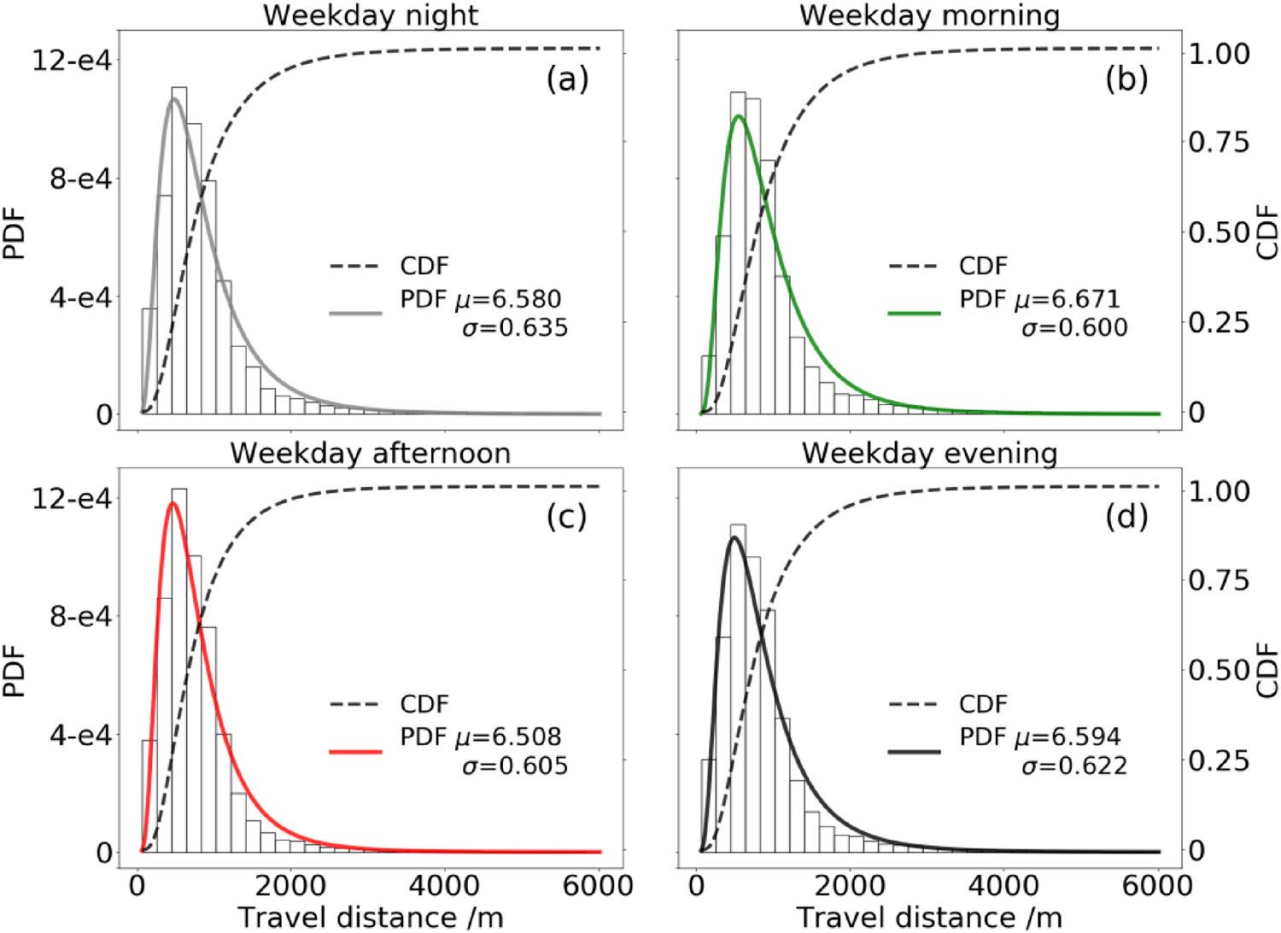
Probability density functions (PDF) and cumulative density functions (CDF) of fitted lognormal distributions for travel distance of FFBS trip within different time ranges on a weekday. The trips with lengths over 6 km (less than 1%) were not plotted here for visualization. (**a**)–(**d**) visualize the results of four periods: nighttime, morning, afternoon, and evening.

The time-varying trips encourage us to investigate the macroscopic spatial features of FFBS usage. The travel distance is selected to apply the probability distribution models to seize the general characteristics of FFBS trips. Human mobility is always empirically observed with a heavy-tailed distribution^44–46^. We use the lognormal distribution to obtain the general characteristics in Fig. 3. This distribution was applied to BSS to test the right-skewed feature of travel distance^11^. The maximum likelihood estimation (MLE) estimates the parameters suggested by Clauset et al.^47^ and Alstott et al.^48^. As a result, trip travel distance fits well in the lognormal distributions, which aligns with a few existing studies^11,49^. Also, as a widely used model for human mobility^44,50^, it is evaluated based on the “*power-law*” package but has lower fitness than the lognormal distribution.

Figure 3 shows the lognormal distribution of trip distances within each period. The distance distribution shows an increasing trend, reaching a peak value at bins 600–800 m. The following decreasing trend is consistent with the spatial decay phenomenon of traveling^51^. The observed distribution of travel distances indicates a notable heterogeneity of cycling behaviors. The distance distributions of trips in different time ranges show a similar characteristic with a slight distinction of statistical variables, including average travel distance $\mu$ and standard deviation $\sigma$. It is counterintuitive because the demands of FFBS and traffic surroundings significantly differ, especially at night and afternoon. The distance of nighttime trips should be shorter and more dispersive due to the unfriendly environment and low usage level. However, it shows an intensive usage of around 600 m, and a proportion of long-distance travel exists. The distribution of travel distance shows the usage stability among periods in a week.

Additionally, long-distance trips exist almost every week, which is not expected for FFBS systems. We attribute these nighttime trips to inevitable travel and the lack of other public transportation services. The daytime, including morning, afternoon, and evening long-distance travel, contains more leisure and sports trips. The distance distributions are generally too simple to distinguish the difference in usage patterns among periods. However, it helps us better understand the spatial distribution of bike-sharing usage and encourages us to explore the temporal-spatial distribution in the next section.

### Spatial comparison of usage patterns among periods

**Figure 4:**
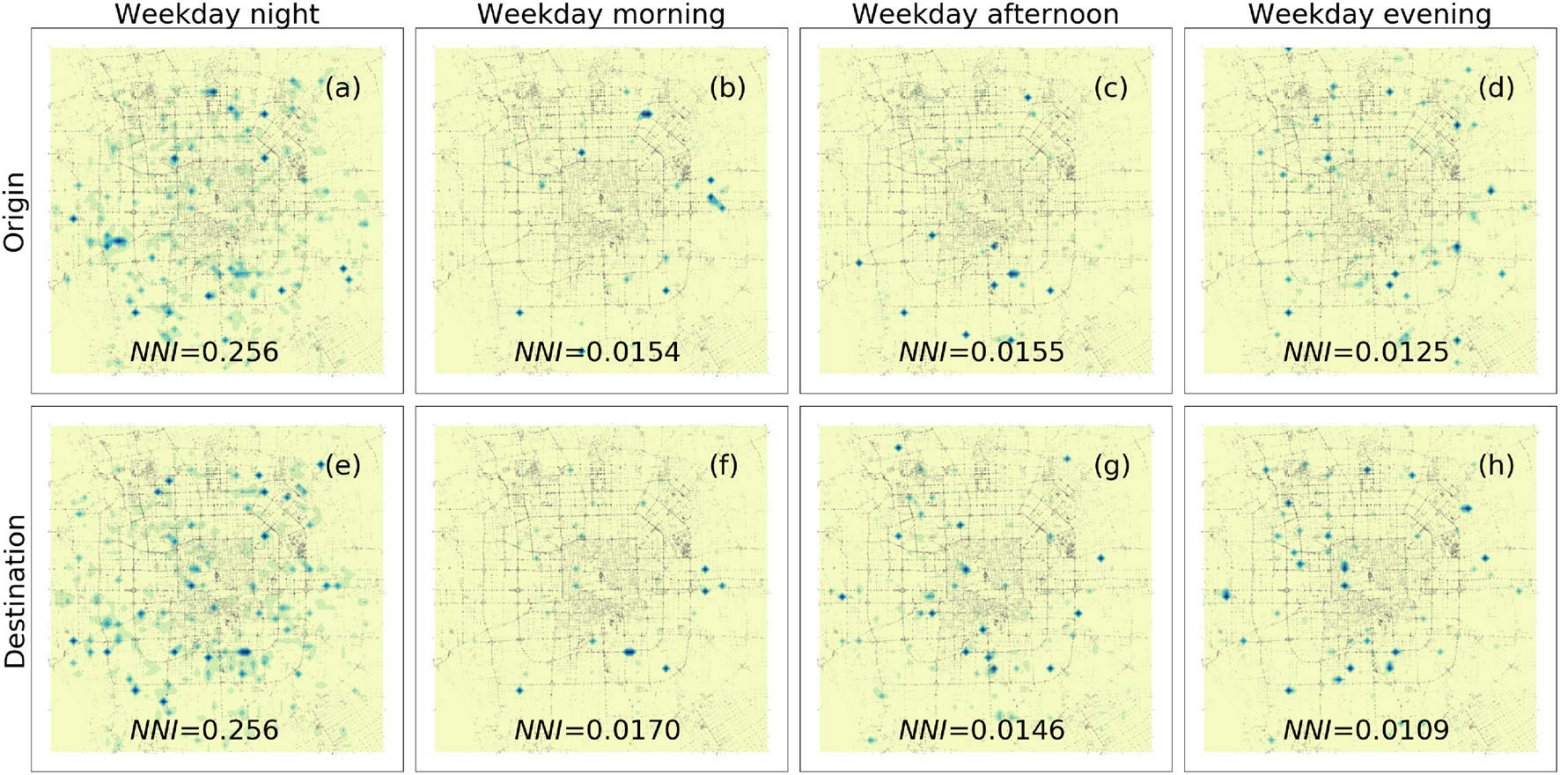
Spatial heatmap of FFBS trips on weekdays. The origins and destinations of trips are separately plotted in subfigures (**a**)–(**d**) and (**e**)–(**h**). The day is divided into four periods: nighttime, morning, afternoon, and evening.

To offer insights into the spatial usage of FFBS among various periods, we apply the sliced spatial heatmap to visualize and capture the patterns, as shown in Fig. 4. The trips are aggregated into the origin and destination views within four periods to compare the usage patterns. The spatial patterns on different time ranges of weekdays and weekends (Detailed information can be found in Figure S4) are also visualized.

The findings offer empirical evidence demonstrating the presence of dissimilar usage patterns of FFBS in different periods. It is observed that the spatial distributions of trip origins and destinations exhibit diverse patterns within the same period. To quantitatively assess the dispersion levels, NNI values from Eq. (1) are depicted in the corresponding panels of Fig. 4. A higher value of NNI means a higher spatial dispersion degree. The usage distributions in views of the origin and destination are close to equilibrium in various periods, consistent with the findings^11^. Specifically, the hotspots of origins and destinations have adjacent distributions consistent with the relative values of NNIs. This similar distribution is reasonable because the FFBS trips have stable distance distributions observed in Fig. 3. It is worth noting that the nearest-neighbor indexes of night spatial distribution are 0.256 ($p<0.01$) in both origins and destinations of trips, indicating that the spatial distribution has prominent agglomeration characteristics. While other spatial usages of FFBS are less than 0.02, suggesting more obvious agglomeration characteristics in the morning, afternoon, and evening periods.

There are more hotspots at night, spreading in different regions of Beijing. The hotpot is a relative comparison over each time range, and the increment of hotpots at night results from the dispersive usage of FFSB, which is more significant on weekend nights in Figure S4. Compared with the usage in the daytime, the nighttime has more hotspots for both origins and destinations of trips, especially in the east, south, and northwest of Beijing, where many employment, leisure, and education places are located. The increment in hotspots is partly due to the decrease in total usage, which reduces usage differences among disparate regions. The usage patterns in the other three periods differ utterly from the nighttime. The low-grade dispersion indicates that the usage of FFBS is concentrated in several areas, mainly in the southeast and west of Beijing. However, the hotspots in the daytime are more likely to become center areas of usage at night, not vice-versa. We attribute this relationship to other social factors like demography and land use. For example, the residential regions may become hotspots of destinations in both daytime and nighttime, while the leisure regions may become the hotspots of origin at night. While the hotspot locations of origins at night in the southwest area marked in the red cycle is a novel place, it does not exist in other periods. In general, the night usage of FFBS is quite different from the pattern observed in other periods.

**Figure 5:**
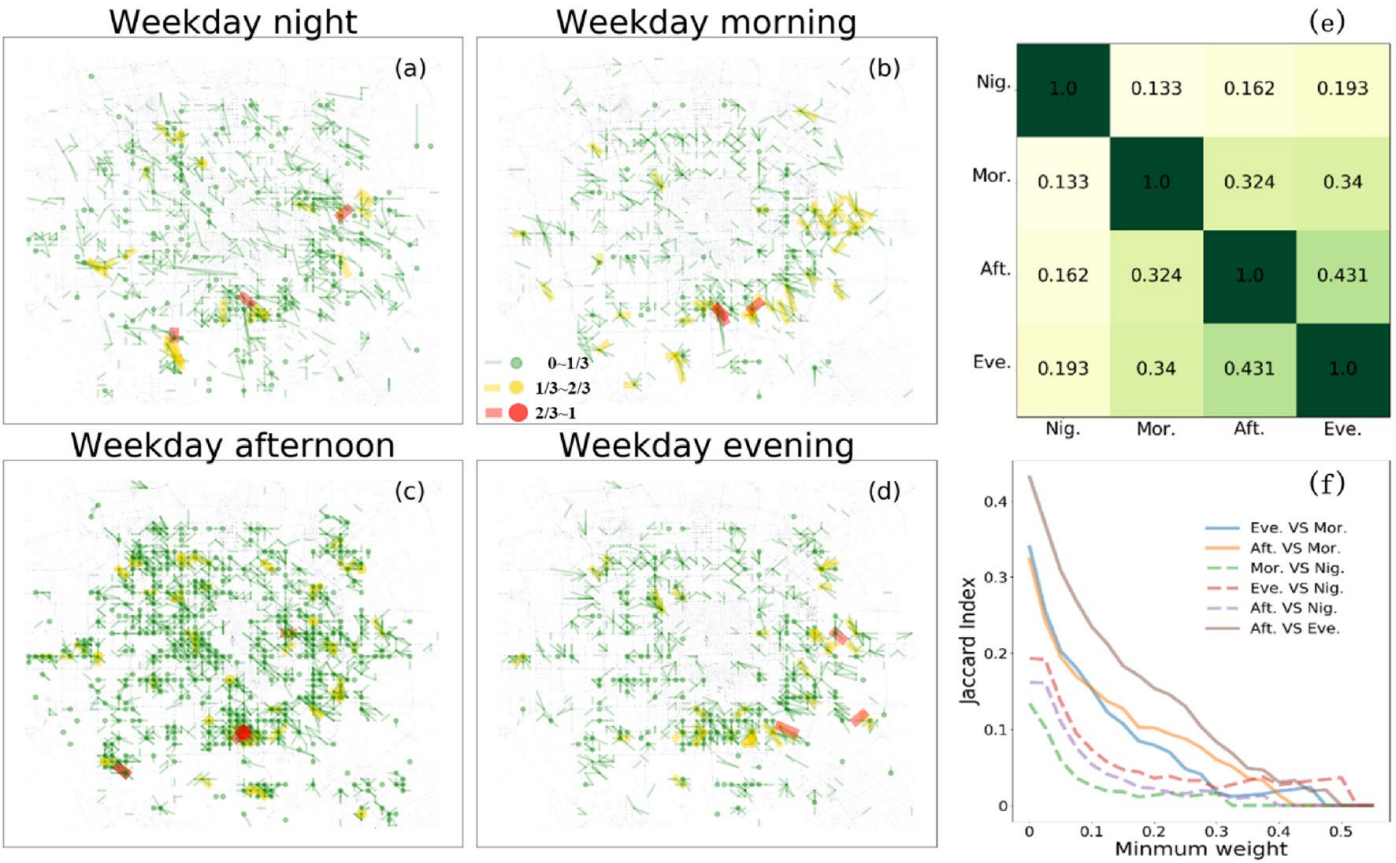
O-D Proportion Flow Graph on Weekdays. (**a**)–(**d**) The ODPFGs with 500m × 500m grid cells in four periods, including nighttime, morning, afternoon, and evening. The maximum trip number normalizes all the connecting edges, and the line width is proportional to the FFBS flow fraction. The size of the dot represents the degree of the nodes. The width of the line represents the weight of the link. It is noted that the proportion flow value is more significant than 0.075. (**e**) The Jaccard index represents the similarity between two ODPEGs. (**f**) The Jaccard index varies with the minimum weight of the edge, where the edges with less weight are removed from the network.

Figure 5 presents the O-D Proportion Flow Graph (ODPFG) derived from the dataset of trips, illustrating the spatial interaction networks across four distinct periods. In this visualization, the size of each point in the graph corresponds to the degree of the respective node, while the width of the colored lines reflects the number of trips connecting the corresponding nodes. The values are normalized by dividing them by the maximum value observed within the corresponding period to ensure a consistent scale for both nodes and links. This normalization enables effective comparison and interpretation of the node and link characteristics across different micro-mobility services.

The analysis of the proposed complex networks reveals significant variations in trip distribution across different periods in Beijing. The nodes with higher degrees, indicative of more substantial trip volumes, are predominantly concentrated in Xicheng, Dongcheng, the southeastern region of Haidian, and the western area of Chaoyang districts. These districts encompass crucial business centers, transportation hubs, educational institutions, and other key locations, as evidenced by the transition from graph (a) to graph (d) in Fig. 5. Furthermore, a comparison of the ODPFGs between weekdays and weekends (see Figure S5) reveals slight disparities, particularly during nighttime on weekends, wherein additional hubs are observed in the southeastern region of Beijing.

To further gauge the similarity and diversity of ODPFGs, we apply the Jaccard index to provide a robust metric for characterizing the difference between ODPFGs in different periods. The distribution of $J$ in Fig. 5e and f shows the distinct usage difference between night and other periods. The FFBS usage pattern at night shares a lower similarity than other periods. However, as Fig. 5e shows, the similarity between the other three periods rises to 0.431. This phenomenon can be attributed to the decreased occurrence of non-essential travel during nighttime, which aligns with the established living habits of the citizens. In Fig. 5f, it is essential to note that although the nodes with higher degrees and the links with greater weights exhibit reduced size and thickness in the connected networks, the mobility distribution of trips remains unchanged between the nighttime and other periods. This observation leads us to speculate that micro-mobility services continue to play a significant role in facilitating human travel during the night despite a decrease in the overall number of trips compared to other periods.

### Demand analysis for FBSS usage with POI

Due to the unbalanced and uneven spatial distribution of FFBS hotspots according to the visualization analysis in the section “Spatial comparison of usage patterns among periods,” we applied the statistical analysis model to recognize the correlation between the usage of FFBS and built-environment elements based on the data of POI. Table 1 presents the estimation results of the zero-inflated negative binomial (ZINB) model, formulated in the “Dataset and Method” section. The parameter coefficients of independent variables have been standardized to ease their relative impact, while z-scores are presented to compare the relative magnitudes of statistical significance. The last two rows in Table 1 show the log-likelihood values for ZINB and ZIP models. It is observed that the log-likelihood values of ZINB models are more significant than those of the ZIP models, declaring the superiority of ZINB models compared with ZIP models for regression fitting based on the collected dataset.

**Table 1:** Regression results in views of trips (OD), origins (O), and destinations (D) of FFBS at nighttime.

Variable	OD		O		D	
	Coef	z-value	Coef	z-value	Coef	z-value
(Intercept)	0.7900	24.704***	0.1982	4.851***	0.1006	2.436**
Entertainment	−	−	0.0588	1.805*	−	−
Food	0.0598	3.508***	0.1091	4.960***	0.1071	4.906***
Employment	0.0709	5.185***	0.0738	4.467***	0.0695	4.214***
Medicine	−	−	−	−	−	−
Shopping	0.1023	6.077***	0.0406	1.995**	0.0866	4.307***
Hotel	0.0741	3.490***	0.0623	2.714***	0.0605	2.646***
Education	0.1045	6.831***	0.0830	4.887***	0.1004	5.967***
Household	0.1137	10.947***	0.0711	6.033***	0.1096	8.566***
Transport (night bus)	0.4333	12.033***	0.5314	14.095***	0.2714	7.066***
Log-likelihood for ZINB model	− 20732		− 13717		− 13883	
Log-likelihood for ZIB model	− 24384		− 18676		− 18642	

***Means significant at the 99% level, ** means significant at the 95% level, * means significant at the 90% level, -means no significant correlation.

Among the considered POIs that may remain available at night, the coefficient of transportation (i.e., the night bus) is the largest among all POIs. This phenomenon proves that the FFBS trips are more likely to become supplementary to the bus at night as a convenient mode for attaching or leaving bus stations to solve the last mile (or the first mile) problem. It is consistent with the function of most FFBS trips in the daytime, which becomes a bike-and-ride for connecting the metro and bus^52^.

Other than transportation factors, the other POIs pertain to an initial origin or final destination of trips rather than a transfer node in trips. Only entertainment and medicine have no significant effect on the usage of FFBS, while other POIs have substantial positive effects. The coefficient of the household is prominent because the household can generate trips to other places and become the destination to go home, which accords with the observation in Ref.^6^. The coefficient of employment is less than the value of the household, which is reasonable since most employment places do not operate at night. One interesting phenomenon is the high positive effect of education, higher than employment and lower than household. This can be attributed to education, such as universities, which simultaneously have similar employment functions (research work) and household (student dormitory) functions. FFBS was first launched in universities in 2017, attracting more demand than communities and urban roads. Another phenomenon is the significant effect of shopping, food, and hotels, but not entertainment. In the previous findings, all these POIs are considered essential leisure places generating mobility at night^53^. It speculates that the requirement of FFBS is low for people who want entertainment places at night. Moreover, although shopping places are mainly out of service at night, shopping places have a similar function and are even adjacent to food places.

As shown in Table 1, the FFBS trips are then divided into origins and destinations to capture the characteristics of flows among various POIs. As expected, transport significantly affects both the origin and destination of trips. However, the coefficient of origin is more extensive than it is for the destination, stating that bus stations generate more FFBS trips rather than attract them. Besides, employment has a higher effect on origins than destinations, while the household has the reverse tendency. This unbalanced effect explains that the FFBS provides a popular mode of transportation for leaving the workplace and going home at night. In the daytime, the opposite flows (i.e., leaving homes or going to work) tend to take another urban transit system, such as the metro. Interestingly, the effect of entertainment becomes significant only with the view of origins. Several FFBS trips are generated from entertainment places rather than going home.

The usage of FFBS during the whole day or the daytime has been investigated, and several primary purposes of trips have been summarized^4,41,54,55^. Six primary purposes are proposed, including dining, transferring (i.e., bike-and-ride), shopping, work-related, going home, and others (i.e., integrated locations)^4^). Table 2 presents the regression results of FFBS trips and POIs concerning four periods to illustrate the variation of the usage pattern throughout a day, which is also consistent with the period division in sections “Time-varying usage and distribution characteristics of travel distance” and “Spatial comparison of usage patterns among periods.” It is important to note that the considered POIs during the three daytime periods have more categories and contents in the same category (see Table S3). More regression results about the characteristics of flows are listed in Table S1.

**Table 2:** Regression results concerning FFBS trips in four periods, including nighttime, morning, afternoon, and evening.

Variable	Nighttime		Morning		Afternoon		Evening	
	Coef	z-value	Coef	z-value	Coef	z-value	Coef	z-value
(Intercept)	0.7900	24.704***	3.2567	143.465***	3.3317	147.439***	3.5921	158.462***
Entertainment	−	−	−0.0654	−2.388**	−	−	−	−
Food	0.0598	3.508***	0.1215	8.014***	0.1136	7.498***	0.1201	7.938***
Employment	0.0709	5.185***	0.0980	7.616***	0.1048	8.152***	0.0679	5.286***
Medicine	−	−	−	−	−	−	−	−
Shopping	0.1023	6.077***	−	−	−	−	0.0265	1.692*
Hotel	0.0741	3.490***	0.0450	2.27**	0.0474	2.394**	−	−
Education	0.1045	6.831***	0.0652	4.726***	0.1362	9.89***	0.0923	6.707***
Household	0.1137	10.947***	0.1413	15.351***	0.1215	13.201***	0.1246	13.553***
Amenity	–	–	− 0.0295	− 1.698*	−	−	−	−
Sport	–	–	0.0462	2.767***	0.0287	1.723*	0.0345	2.071**
Leisure and travel	–	–	0.0213	1.689*	0.0283	2.244**	−	−
Transport (metro)	–	–	0.5365	6.238***	0.4397	5.113***	0.3960	4.608***
Transport (bus)	–	–	0.4196	38.177***	0.3808	34.653***	0.3675	33.464***
Transport (night bus)	0.4333	12.033***	–	–	–	–	–	–

***Means significant at the 99% level, ** means significant at the 95% level, * means significant at the 90% level, -means no significant correlation.

Congruent to existing research (e.g., Böcker et al.^41^), urban transportation services, including the metro and bus, are significant factors for FFBS usage over all periods. The metro has a more substantial positive effect than the bus in the daytime. The positive effects of household and employment places are also observed in Böcker et al.^41^, where a higher density of jobs leads to more destinations for FFBS trips. Food is a widely uncovered factor that improves the usage of FFBS, surpassing other land use characteristics^55^. Trips related to dining activities account for the maximum proportion of conjectural trips^4^. Education is also recognized as a critical factor in the usage of FFBS, which is always overlooked in the existing research. Besides, several POIs, such as entertainment, medicine, amenity, leisure, and travel, have almost no significant relationship with FFBS usage.

Generally, there are several similarities between the patterns of POIs in the four periods. For example, transport, education, food, and household show a stable, increasing effect on FFBS usage in different periods. The night bus tends to become the origin of the trips, similar to the metro but different from the day bus. A noticeable difference is employment, which attracts more morning and afternoon trips while generating more in the evening and nighttime. This phenomenon reveals that FFBS is a common way to make work-related trips at night. Another difference is shopping, the primary purpose of FFBS estimated by Wang et al.^55^. The effect of shopping is not apparent in the daytime (especially in the morning and afternoon) but significant at night. We attribute this difference to the concentrated distribution of shopping places and the small size of grid cells for analysis. Based on the above analysis of the FFBS trips within a week, the differences observed in sections “Time-varying usage and distribution characteristics of travel distance” and “Spatial comparison of usage patterns among periods” compel us to analyze the usage patterns of FFBS during weekday and weekend nights.

The average number of trips per day with decimals cannot be applied in the ZINB, which requests the integral dependent variables (i.e., the number of trips). Therefore, the number of trips is multiplied by 2 for weekends and 5 for weekends for an equivalent quantitative comparison. Table 3 illustrates the estimation results based on the nighttime trips on weekdays and weekends. More estimation results with origin or destination view are provided in Table S2.

**Table 3:** Regression results of FFBS during the nights on weekdays and weekends.

Variable	weekday night (OD)		weekend night (OD)	
	Coef	z-value	Coef	z-value
(Intercept)	1.1239	34.646***	1.13114	33.382***
Entertainment	−	−	−	−
Food	0.0734	4.200***	0.1418	6.394***
Employment	0.0671	4.842***	0.0897	5.138***
Medicine	−	−	−	−
Shopping	0.0827	4.838***	0.0426	2.077**
Hotel	0.0745	3.569***	0.0464	2.085**
Education	0.1074	7.103***	0.0727	4.458***
Household	0.1065	10.138***	0.0842	6.599***
Transport (night bus)	0.4080	11.476***	0.4556	12.639***

***Means significant at the 99% level, ** means significant at the 95% level, * means significant at the 90% level, -means no significant correlation.

A larger transport coefficient with a more significant effect on weekend nights is uncovered, consistent with Fig. 1, that more trips are observed on Friday and Saturday nights. A visible difference is the coefficient increment about food on weekend nights, reflecting that people have more time for dinner or are likelier to take night snacks. The employment coefficient is higher on weekend nights, which is counterintuitive since the number of people working on day offs has naturally dropped. However, this remarkable decrement mainly occurs in the daytime rather than the nighttime. This result suggests that more trips are generated from employment due to more working people on weekend nights. The coefficient increment about food and employment can partly support Fig. 4, especially in Beijing's east and northeast areas.

The POIs of shopping, hotels, education, and households have similar decrement variations. Education, which has both functions of household and employment, has a more similar characteristic to a household. These results are consistent with the findings in Table 1. The hotel is significant in the single view of origins and destinations only weekly (see Table S2), reducing the coefficient. Additionally, shopping is more significant for FFBS usage only as the weekday destination.

## Discussion and conclusion

FFBS has developed rapidly with its convenience, health, and flexibility, attracting more users, primarily used for short-distance mobility and bike-and-ride trips^56,57^. Understanding the variation of FFBS usage patterns and the effects of a non-linear built environment can help traffic managers initiate appropriate measures at the planning stages by identifying usage distribution, source of demand, and its relationship with urban transit systems^58,59^. The nighttime also provides an excellent time window to observe the relationship between FFBS and other transit systems, such as the night buses. The FFBS is a superior option for unavoidable nighttime travel demand because of its cost-efficient expense and easy accessibility when most urban transit systems are unavailable.

To intuitively observe the variation of FFBS usage patterns, we divide each day into four typical periods, including night, morning, afternoon, and evening, and the nighttime is the focus period of this paper. The temporal-spatial analyses are first conducted to investigate the usage patterns of FFBS at various time ranges. The temporal characteristics are illustrated in views of additional half-hours and different nights, and imbalanced spatial usage distribution in different periods indicates the various mobility patterns. We then concentrate on correlations between different periods by comparing the O-D flow of FFBS. Finally, the usage of FFBS is explored according to a zero-inflated negative binomial statistical model. The main findings are summarized below:

I. Besides the public knowledge of the morning and evening perk trend, the temporal usage on various nights has a similar U-shaped variation with abrupt changes on both sides, which implies the wide usage of FFBS for connecting to the last or first urban transit systems. The usage of FFBS on weekend nights is more significant than on weekday nights, although the number of trips throughout the day on a weekday is higher than that on the weekend. Although there are differences in the amount of FFBS usage in various periods, the distance of trips presents a stable distribution.
II. From a spatial perspective, the usage of FFBS at night is significantly more dispersive than in daytime periods, as evidenced by the NNI index and ODPFG similarity measurement. The hotspots during the daytime are more concentrated and tend to become hotspots at night. The nighttime has more scattered hotspots, especially in areas with employment, leisure, food, education, etc.
III. The relationship between FFBS and multi-mode urban transportation is summarized. The night bus has a strong favorable attraction to FFBS and has become the origin of trips, similar to the metro. The day bus has a significant and balanced impact on FFBS usage. As a result, the FFBS tends to be a mode linking the night bus and metro to solve the last-mile problem.
IV. The correlation analysis between FFBS and POIs reveals the source of FFBS demands. The FFBS is positively encouraged by the POIs, who may still be available at night except for medicine. The impacts of household, shopping, and education are relatively significant, and these locations are often the destinations for FFBS trips. Besides transport, places with food and employment have a higher impact on FFBS on weekday nights.

Our results raise awareness of the variation in usage patterns of FFBS by focusing on the night pattern and the difference between nighttime and daytime. It can benefit various applications, such as transportation planning and urban management, in deploying free-floating bike sharing according to complicated travel requirements. The valuable information provided by bike sharing makes it possible to distinguish the hotspots of nighttime travel and accurately plan new night bus lines, mainly relying on human surveys^60^. The correlation between FFBS and POI proposes a new perspective for the coordinated development of transport and land use, emphasizing the time-varying relationship between travel demand and land types.

While the results are exciting, the paper leaves several limitations owing to the datasets and approaches to be desired. First, due to the availability of the dataset, possible biases may occur in catching mobility patterns from one FFBS company (i.e., Mobike). Secondly, only a 7-day FFSB dataset is used in the analysis process, which limits the long-term usage pattern variation and comparison, such as the seasonal changes. The dataset with more sources and longer time ranges helps enhance the generality of findings. Thirdly, while the statistical analysis of FFBS and POIs reveals the built-environmental effect on the usage, it cannot figure out the purpose of travel, as there may be many possible activities in the grid cell with various POIs. A more accurate dataset of POI is required to elaborately identify the purpose of the trip and improve the practical application of this paper.

## Dataset and Method

This section begins with various datasets collected from Beijing, including public transportation networks, points of interest, and trip data obtained from Mobike. Then, a series of methods for data processing, statistical analysis, and visualization of results are put forward.

### Study area

**Figure 6:**
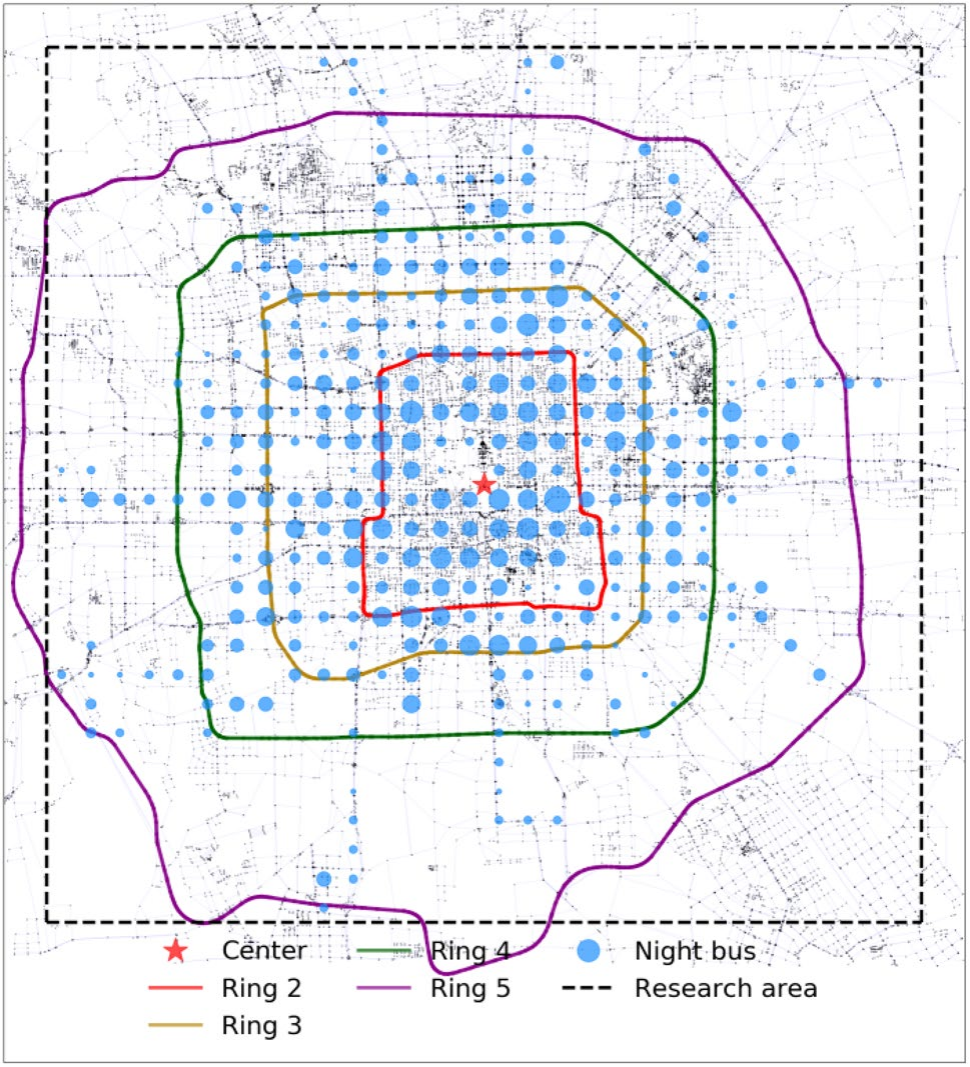
The research area in Beijing. Rings 2–5 are bolded with red, brown, green, and purple colors. The blue circle indicates the service frequency of the night bus within the nearby area.

Beijing is one of the typical big cities in the world, with a population of more than 21 million and an administrative area of 16,410 ${\text{km}}^{2}$. Rapid urban roads enclose the central area of Beijing, namely Rings 2–5. As shown in Fig. 6, the adopted research area is a square with a size of 30 $\times$ 30 ${\text{km}}^{2}$ centered by Tian'anmen, mainly covering the urban area within Ring 5.

### Free-floating bike sharing dataset

The data of FFBS from Mobike company contains the records of trips within a week from 07/10/2017 (Wednesday) to 07/16/2017 (Tuesday). The records include 1,830,101 trips with O-D locations and start times generated from 400,000 bikes. Each record has the following attributes: User ID, Bike ID, Check-in Longitude, Check-in Latitude, Check-out Longitude, Check-out Latitude, and start time. It is essential to note that the drop-off time (i.e., the end time) is not collected.

At the preprocessing stage for data cleaning, the valid FFBS trips are selected with complete details and no abnormal information and travel distances ranging from 0.1 to 15.0 km^9,34^.

### Point of interest dataset

A point of interest (POI) is a specific location where people may search for or visit. The POIs near the end of the trip can reveal the likely purpose of the travel^61^. Combining FFBS and POIs datasets can effectively reveal the FFBS demand within various geographic features^8^. The POI data collected from BaiduMap is distinguished for POIs in service at night. These night POIs are classified into nine categories in Table 4, where several common POIs, such as sports and amenities, are deemed not in service^56^. Urban transportation at night (i.e., the night bus) constitutes the transport categories, which are visualized in Fig. 6. The available POIs on the day or night are diverse and have been carefully distinguished by opening time or categories. The information on available POIs on the day is provided in Table S3. The distribution of several typical POIs is visualized in Figures S6–S10, which is associated with the distribution of bike-sharing usage.

**Table 4:** Categories and classification of available POIs at night.

POI categories	Specific details in categories
Entertainment	Bar, KTV, Teahouse, Internet cafe, Cinema, Dancing hall, Gaming
Food	Restaurant (local and foreign), Snake, Cake, Cafe
Employment	Company, Factory, Government, Office
Medicine	Hospital, Polyclinic, Clinic, Drugstore
Shopping	Emporium, Convenience store, Shop, Store
Hotel	Hotel, Budget hotel, Inn, Homestay
Education	University, Scientific institution
Household	Residential building, Dormitory
Transport	Night bus station

### Grid cell division

The free-floating mobility of FFBS generates the self-organized distribution of bikes over the cities^18^. The research area is partitioned into homogeneous square grid cells to facilitate the statistics and analysis. It is worth mentioning that the longitude and latitude coordinates are transformed from the World Geodetic System to a projected system (Gauss-Kruger) before building the grid cell.

For different analytical purposes, we aggregate FFBS trips into grid cells in intra-cell or inter-cell ways. First, the number of intra-cell trips starting and ending within cell $i$ are respectively aggregated as ${s}_{i}^{O}$ and ${s}_{i}^{D}$. The demand related to cell $i$ is further denoted as ${s}_{i}^{OD}$, and ${\text{s}}_{i}^{OD}={s}_{i}^{O}+{s}_{i}^{D}$. Then, the inter-cell travel flow between cells $i$ and $j$ is represented by ${s}_{ij}$ and the remaining trips whose origin and destination exist in one cell $i$ are aggregated as ${s}_{i}$. In general, more giant cells transfer more FFBS trips inside the cells, while a small style division improves the accuracy of describing the FFBS flows but increases connections between non-adjacent cells. In this study, the size of grid cells depends on the analytical purposes and precision of the dataset.

### Spatial aggregation characteristics of hotspot pattern

Spatial aggregation is one of the critical features of the FFBS usage patterns. A point pattern approach is applied to examine whether the FFBS trips tend to cluster or randomly spread across the city area, i.e., the hotspot pattern. To perform the hotspot pattern analysis of the spatial aggregation of FFBS usage quantitatively, we implemented a Nearest-Neighbor Index (NNI) method.

The $NNI$ is counted to judge whether the distribution factors of points are scattering or clustering. These points include the origin and destination coordinates of all trips. The core of the method is comparing the average distance of nearest neighbor point pairs and that under random distribution conditions^62^. The $NNI$ is calculated as:

1 $$NNI=\frac{\sqrt{A}{\sum }_{i=1}^{n}{d}_{i}}{2\sqrt{{n}^{3}}},$$

where ${d}_{i}$ is the shortest distance between point $i$ and other points, $n$ is the total number of points, and $A$ is the area of a minimum enclosing rectangle around all points.

As a result, the FFBS usage has a clustered pattern when $NNI$ values close to 0 and tends to be a random or scattering pattern when $NNI$ values close to 1. The pattern with the $NNI$ values larger than 1 means a uniform distribution mode.

### Spatial structural characteristics of mobility pattern

Despite the spatial aggregation characteristics depicting the usage hotspots of the FFBS in terms of trip origins and destinations, a comprehensive understanding of the inherent travel characteristics in these usage patterns remains lacking. Therefore, we delve into the mobility pattern, which encompasses the flow between different spatial grid cells, providing valuable insights into the spatial–temporal dynamics of FFBS usage. A dedicated tool, the O-D Proportion Flow Graph (ODPFG), is developed to facilitate analysis and visualization. Within the context of FFBS, the mobility pattern refers to the movement between cells, categorized as intra-cell or inter-cell flow. By adopting the ODPFG, we present a concise yet informative visualization method for comprehending the flow patterns associated with FFBS usage.

The O-D Proportion Flow Graph (ODPFG) is a conventional undirected flow graph portraying linear mobility patterns between grid cells. Comprising nodes and links, the ODPFG represents the connectivity between cells, with the line (or link) denoting the linkage between two distinct cells (nodes). The width of the line in the ODPFG corresponds to the strength or intensity of the connectivity, effectively reflecting the magnitude of flow between the cells. Specifically, the link weights in the ODPFG are derived from the FFBS flows, signifying the usage intensity associated with the respective connections.

To explore the structural properties of ODPFGs, we utilize the Jaccard coefficient to investigate the similarity between two arbitrary ODPFGs through the local structural property. To this end, the trips in one period from one cell to another are selected and compared with those in another period. We apply the Jaccard similarity coefficient^63^ ($J$) and can be calculated as

2 $$J\left(R\left(i\right), R\left(j\right)\right)=\frac{\left|R\left(i\right)\cap R\left(j\right)\right|}{\left|R\left(i\right)\cup R\left(j\right)\right|},$$

where $R$ is the vector of weighted edges in the directed graph, and $i$ ($j$) represents the period.

### Statistical correlation analysis

To identify the geographical and built-environment factors that can potentially generate or attract FFBS trips, we use a multivariable modeling technique to analyze the relationship between the usage of FFBS and POIs. The data of FFBS and POIs were divided into the 300 m $\times$ 300 m grid cells corresponding to the precision of POI data.

In the statistical model, let ${X}_{i}$ and ${Y}_{i}$ denote the independent variable vector and the dependent variable of cell $i,$ respectively. The dependent variables are different types of FFBS usage about a cell, which can be among ${s}_{i}^{O}$, ${s}_{i}^{D}$ and ${s}_{i}^{OD}$. The independent variables are the number of POIs corresponding to each category. More statistical information about POIs in cells is provided in Table S4. Since the dependent variables contain a large proportion of zeros, we adopted the zero-inflated negative binomial (ZINB) regression to model the usage of FFBS^55^. The ZINB model can effectively address two issues from the datasets. Firstly, a large proportion of zeros occurs in POI data with a finer spatial granularity, where the ZINB model can aptly account for the excessive zeroes in the independent variables. Secondly, the dependent variable (i.e., the count of bike-sharing usage) is discrete, and the binomial regression in ZINB can effectively capture the characteristic information about the dependent variables.

In ZINB regression, ${Y}_{i}$ specifically follows a zero distribution with a probability ${p}_{i}$ and a negative binomial distribution (NBD) with probability $(1-{p}_{i})$:

3 $$P\left({Y}_{i}={y}_{i}\right)\sim \left\{\begin{array}{cc}{p}_{i}+\left(1-{p}_{i}\right){\left(1+\frac{{\tau }_{i}}{{\lambda }_{i}}\right)}^{-{\lambda }_{i}}& {y}_{i}=0\\ \left(1-{p}_{i}\right)\frac{\Gamma \left({y}_{i}+{\lambda }_{i}\right)}{{y}_{i}!\Gamma \left({\lambda }_{i}\right)}{\left(1+\frac{{\tau }_{i}}{{\lambda }_{i}}\right)}^{-{\lambda }_{i}}{\left(1+\frac{{\lambda }_{i}}{{\tau }_{i}}\right)}^{-{y}_{i}}& {y}_{i}=\text{1,2}\dots \end{array}\right.,$$

where probability ${p}_{i}$ is calculated from a logit distribution based on independent variables:

4 $$logit \left({p}_{i}\right)={X}_{i}{\prime}\alpha ,$$

and ${\tau }_{i}$ is the mean value of ${Y}_{i}$ in the NBD:

5 $$\mathit{ln}\left({\tau }_{i}\right)={X}_{i}{\prime}\beta ,$$

and ${\lambda }_{i}$ is the dispersion parameter of NBD. The ZINB distribution will reduce to a Zero-inflated Poisson (ZIP) distribution if ${\lambda }_{i}\to \infty$, which is a widely applied model for relationship analysis.

The maximum likelihood estimation (MLE) method is applied to select the $\alpha$ and $\beta$ with the best fit for the ZINP regression. A natural logarithm transformation on independent variables is better for model fit^54,56^. Due to many zeros in the independent variables caused by no POIs belonging to one category existing in small grid cells, a linear transformation from ${X}_{i}$ to ${X}_{i}+1$ is performed before the logarithm transformation to avoid the undefined error.

### Supplementary Information

Supplementary Information.

## Data Availability

The datasets generated during and/or analyzed during the current study are available from the corresponding author upon reasonable request.
