# Supplementary material for: Exploring usage pattern variation of free-floating bike-sharing from a night travel perspective
Source: Sci Rep. 2024 Jul 11;14:16017. doi: 10.1038/s41598-024-66564-2 (PMC11239850; doi:10.1038/s41598-024-66564-2)
Supplement: Supplementary file 1 — Supplementary Information. [file 41598_2024_66564_MOESM1_ESM.pdf]

Exploring usage pattern variation of free-floating bike-sharing from a night travel perspective

Supplementary materials

Supplementary Figure S1. Rescaled time-varying trips per 30 minutes N/N_{max} within a week from 05/10/2017 (Wednesday) to 05/16/2017 (Tuesday), where N is the number of trips and N_{max} is the maximum number of trips for all the time individuals. The light gray areas indicate the nighttime from 23:00 to 5:00.

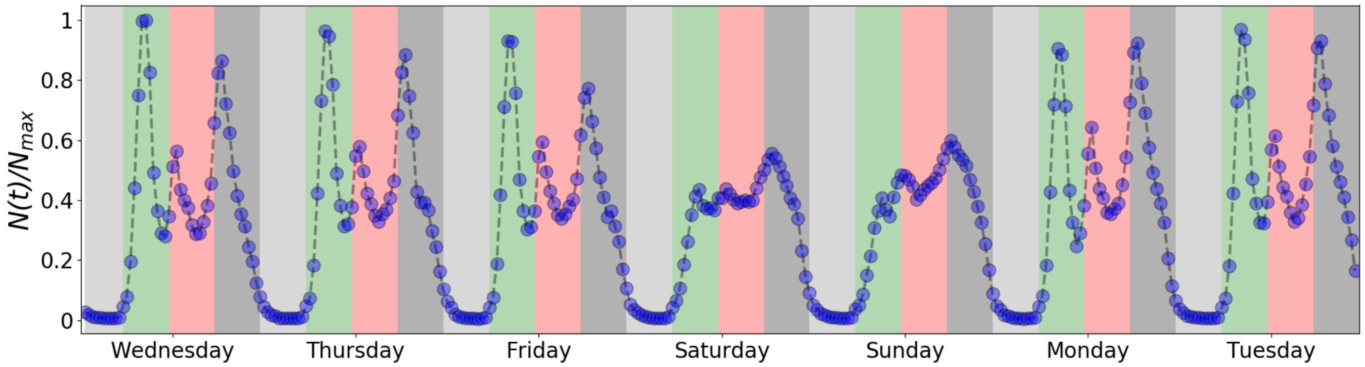

Supplementary Figure S2. The probability of usage frequency in a week among four periods.

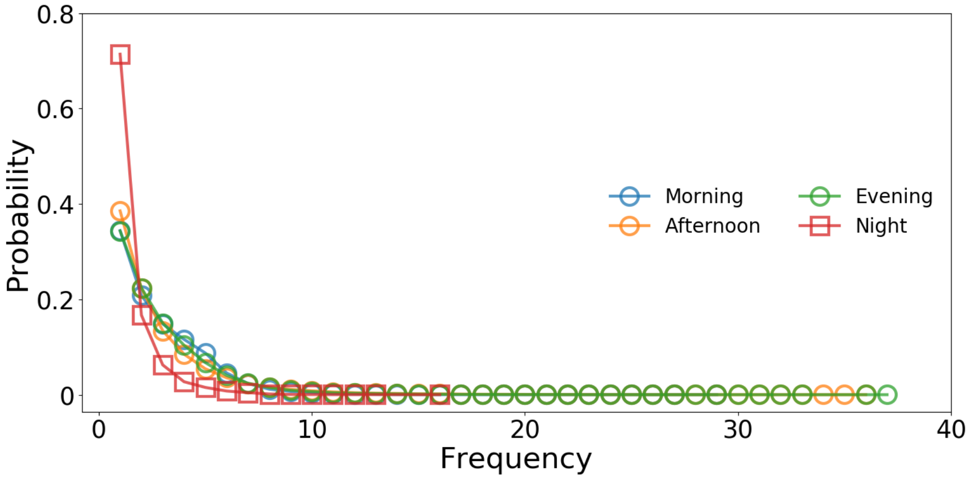

Supplementary Figure S3. Probability density functions (PDF) and cumulative density functions (CDF) of fitted lognormal distributions for travel distance of FFBS trips within different periods. The trips with lengths over 6 km (less than 1%) were not plotted here for visualization.

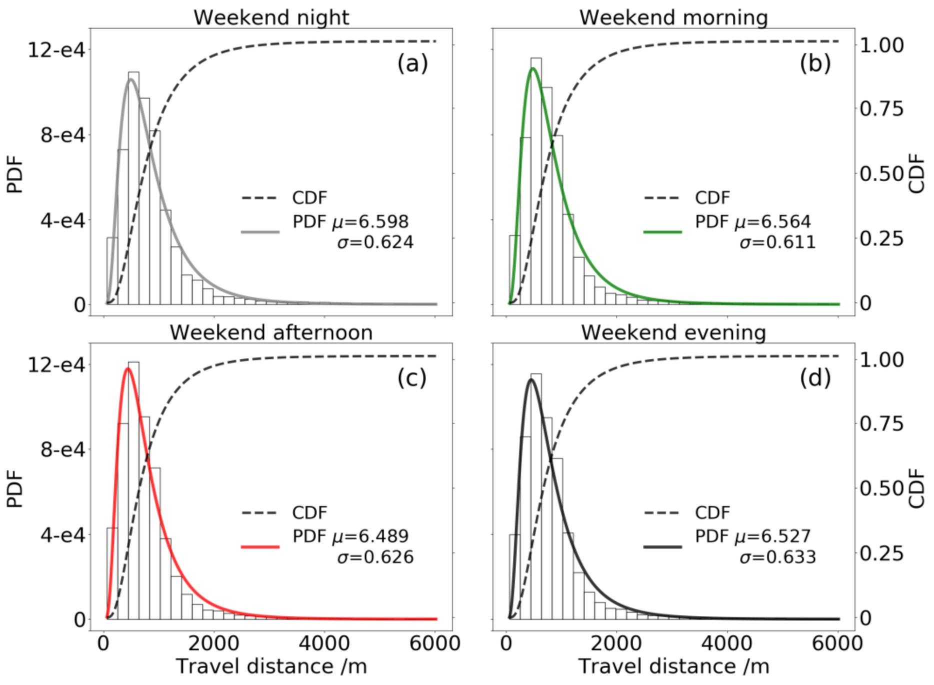

Supplementary Figure S4. Spatial heatmap of FFBS trips in various periods. The origins and destinations are separately plotted in subfigures (a)-(d) and subfigures (e)-(h). The day is divided into nighttime (23:00 - 5:00) and daytime (5:00 - 23:00).

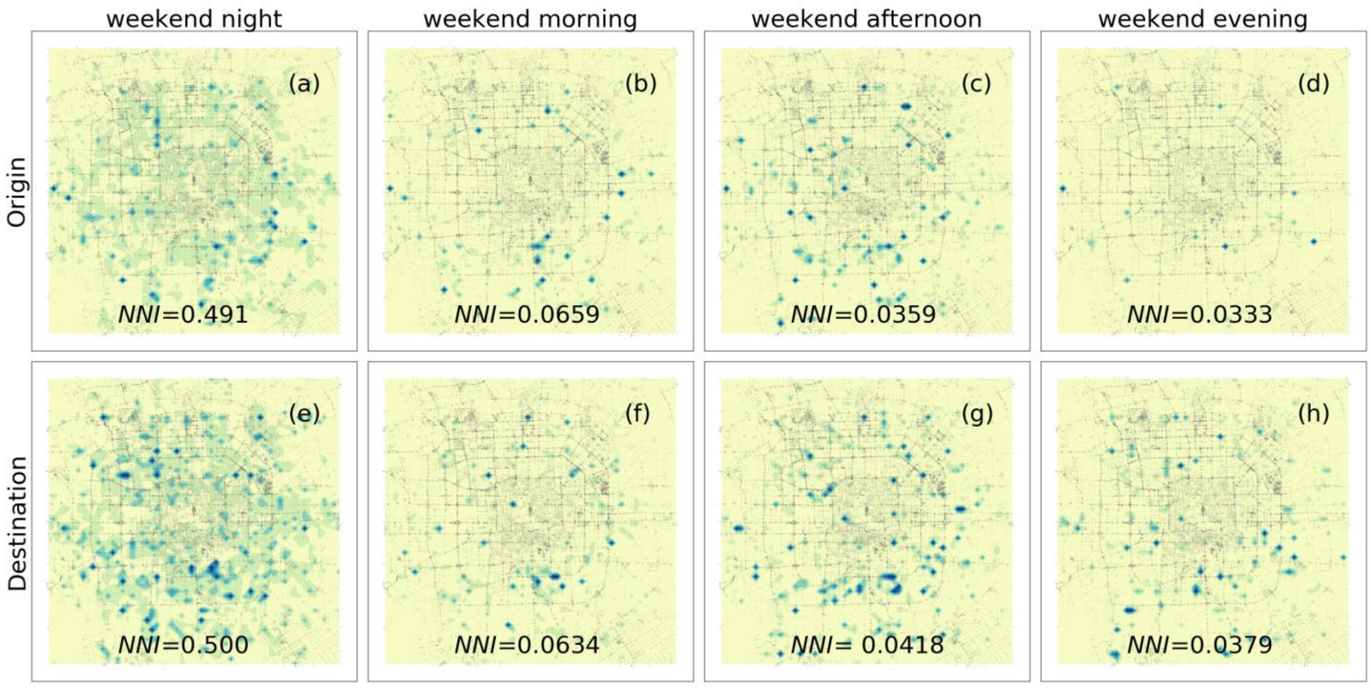

Supplementary Figure S5. O-D Proportion flow graph on weekdays. The trips' origins and destinations are plotted separately from subfigure (a) to subfigure (d).

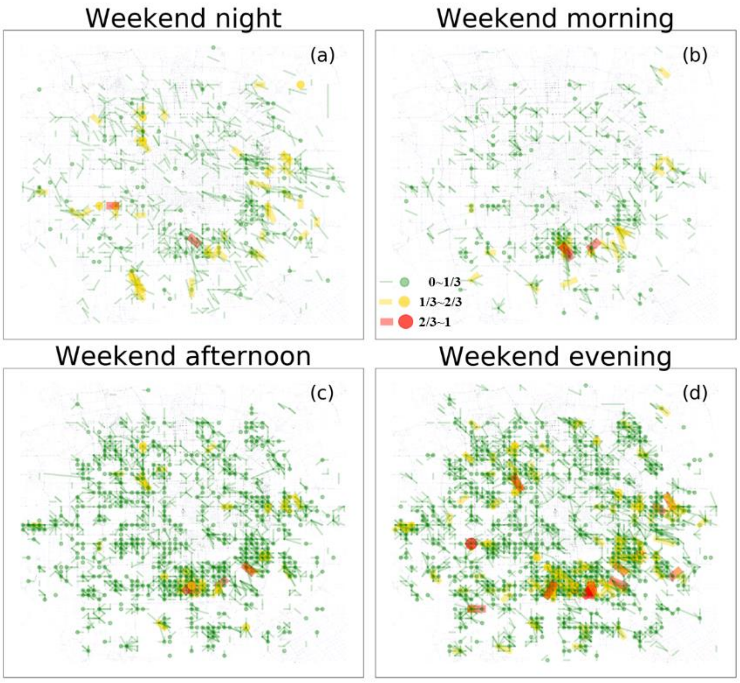

Supplementary Figure S6. Spatial heatmap of education POI at day and night.

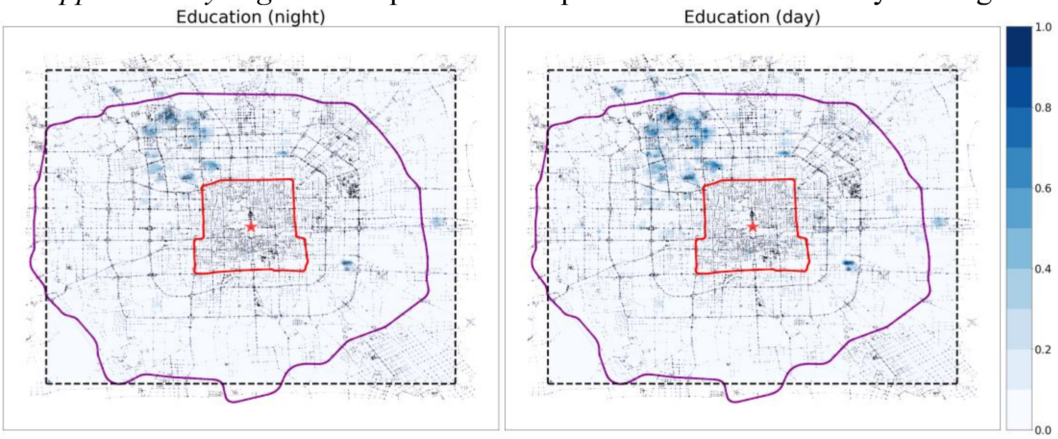

Supplementary Figure S7. Spatial heatmap of employment POI at day and night.

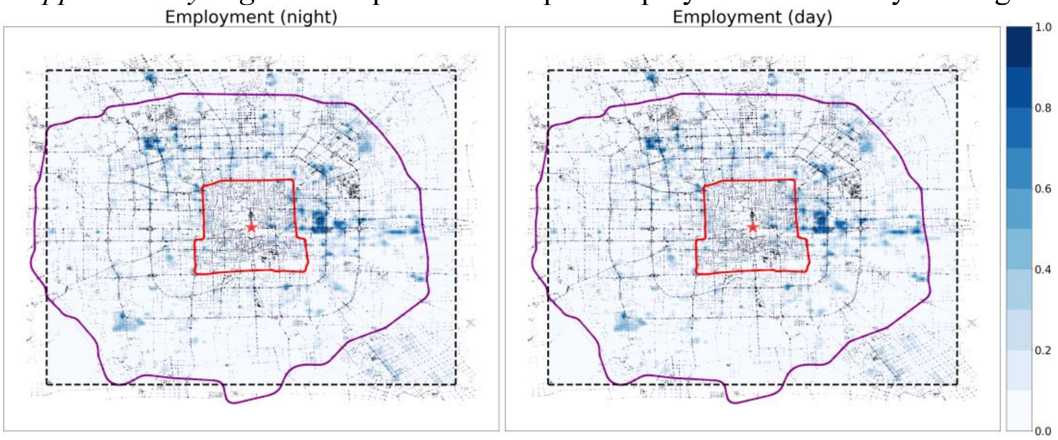

Supplementary Figure S8. Spatial heatmap of entertainment POI at day and night.

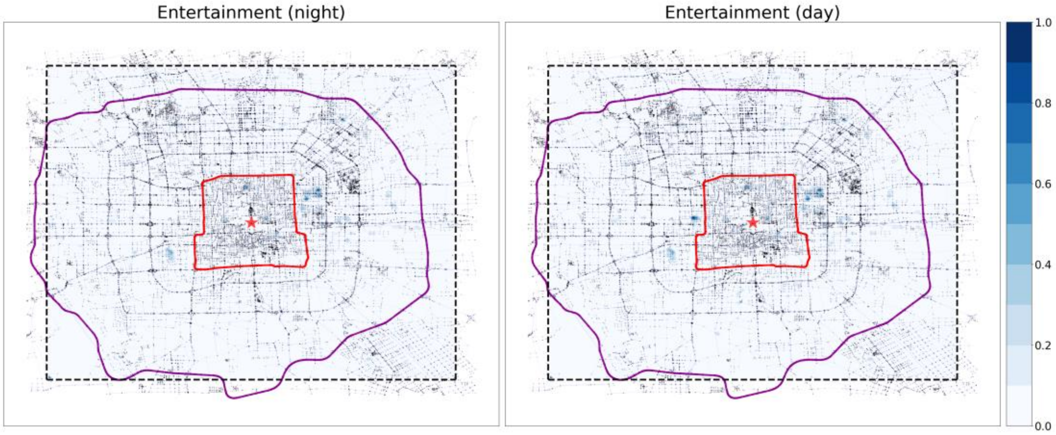

Supplementary Figure S9. Spatial heatmap of shopping POI at day and night.

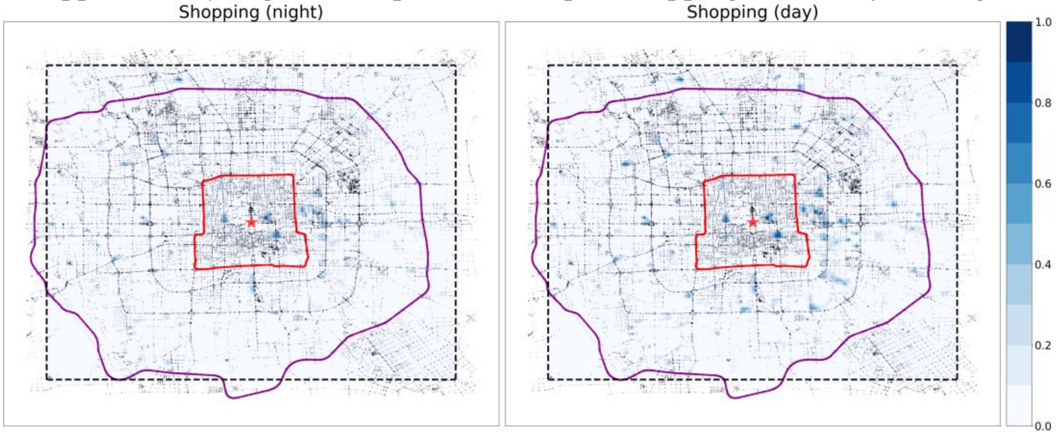

Supplementary Figure S10. Spatial heatmap of household and hotel POI.

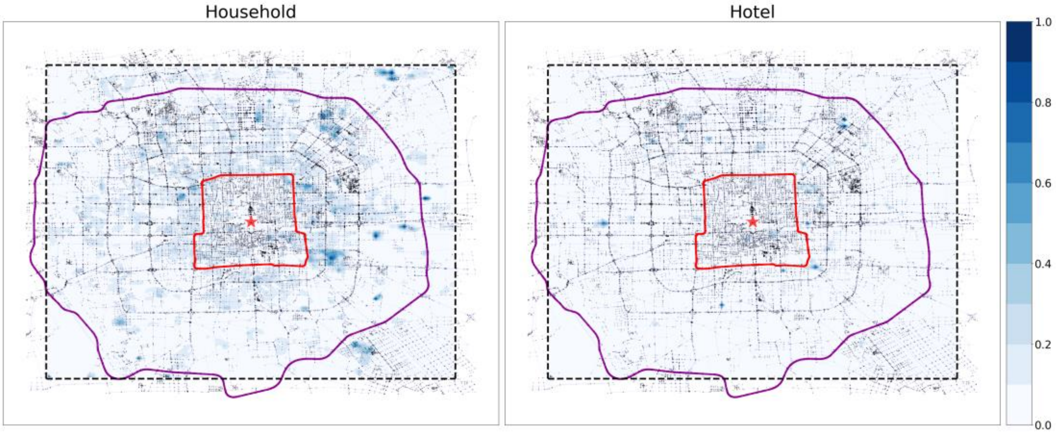

Supplementary Table S1. Regression results in views of origins (O) and destinations (D) of FFBS trips concerning four periods: nighttime, morning, afternoon, and evening.

Variable	Nighttime		Morning		Afternoon		Evening	
	O	D	O	D	O	D	O	D
(Intercept)	0.198***	0.101**	2.723***	2.427***	2.681***	2.612***	2.886***	2.937***
Entertainment	0.059*	-	0.057**	0.074***	-	-	-	-
Food	0.109***	0.107***	0.108***	0.127***	0.108***	0.116***	0.124***	0.110***
Employment	0.074***	0.070***	0.054***	0.135***	0.098***	0.108***	0.084***	0.047***
Medicine	-	-	-	-	-	-	-	-
Shopping	0.041**	0.087***	-	-	-	-	-	0.050***
Hotel	0.026***	0.061***	0.065***	-	0.043**	0.053***	-	-

Education	0.083***	0.100***	0.046***	0.081***	0.125***	0.147***	0.092***	0.092***
Household	0.077***	0.110***	0.141***	0.139***	0.117***	0.125***	0.106***	0.140***
Amenity	/	/	-	-0.039**	-	-0.032*	-	-
Sport	/	/	0.054***	0.038**	0.030*	-	0.038**	0.030*
Leisure and travel	/	/	0.030**	0.035***	0.039***	0.024*	-	-
Transport (metro)	/	/	0.557***	0.502***	0.553***	0.305***	0.547***	0.213**
Transport (bus)	/	/	0.356***	0.478***	0.387***	0.371***	0.413***	0.317***
Transport (night bus)	0.5314***	0.2714***	/	/	/	/	/	/

Note: *** means significant at the 99% level, ** means significant at the 95% level, * means significant at the 90% level, - means no significant correlation.

Supplementary Table S2. Regression results in views of origins (O) and destinations (D) of FFBS trips on weekdays and weekend nights

Variable	weekday night (O)		weekday night (D)		weekend night (O)		weekend night (D)	
	Coef.	z-value	Coef.	z-value	Coef.	z-value	Coef.	z-value
(Intercept)	0.5628	12.978***	4.919	11.340***	0.7498	11.453***	0.7290	11.639***
Entertainment	0.0857	2.550**	-	-	0.1633	3.149***	-	-
Food	0.1242	4.970***	0.1269	5.158***	0.1113	2.694***	0.1740	4.636***
Employment	0.0562	2.945***	0.0778	4.149***	-	-	0.0582	1.677*
Medicine	-	-	-	-	-	-	-	-
Shopping	-	-	0.0515	2.294**	-	-	-	-
Hotel	0.0628	2.594***	0.0444	1.860*	-	-	-	-
Education	0.0951	5.355***	0.0973	5.566***	-	-	-	-
Household	0.0727	5.095***	0.0973	6.910***	0.1249	5.129***	0.1301	6.128***
Transport	0.4960	13.057***	0.2247	5.867***	0.6531	10.976***	0.2725	5.442***

Note: *** means significant at the 99% level, ** means significant at the 95% level, * means significant at the 90% level, - means no significant correlation.

Supplementary Table S3. Categories and classification of available POIs in the day

POI categories	Specific details in categories
Entertainment	Resort, Agritainment, Theater, Bar, KTV, Teahouse, Internet cafe, Cinema, Dancing hall, Gaming
Food	Restaurant (local and foreign), Snake, Cake, Cafe
Employment	Art group, Broadcast, Press, Publisher, Company, Factory, Government, Office
Medicine	Sanatorium, Centers for Disease Control, Medical apparatus, Health, Hospital, Polyclinic, Clinic, Drugstore
Shopping	Beauty salon, Manicure, Home appliance, Build material, Wholesale, Emporium, Convenience store, Shop, Store
Hotel	Hotel, Budget hotel, Inn, Homestay
Education	Primary/secondary school, Kindergarten, Adult education, Science museum, University, Scientific institution
Amenity	Post office, Intermediary, Housekeeping, News kiosk, Public toilet, Car service, Communication, Pet service
Sport	Fitness, Gymnasium, Extreme sports
Leisure and travel	Park, Zoo, Amusement, Cultural relics, Temple, Beauty Spot, Leisure square, Massage bath
Household	Residential building, Dormitory
Transport	Metro station, Day bus station

Supplementary Table S4. Descriptive statistics of POIs within grid cells in the day and night. The number of POIs in day categories contains the number in night categories.

POI	Day	Night

	min	max	mean	std. deviation	min	max	mean	std. deviation
Amenity	0	290	5.231	9.578	-	-	-	-
Education	0	126	3.854	8.771	0	93	1.693	6.188
Employment	0	203	11.166	21.971	0	198	10.910	21.557
Entertainment	0	30	0.440	1.284	0	23	0.386	1.153
Food	0	144	8.335	11.696	0	117	5.179	9.265
Hotel	0	60	0.949	2.709	0	60	0.949	2.709
Household	0	300	16.723	26.159	0	300	16.723	26.159
Leisure and travel	0	222	2.597	8.025	-	-	-	-
Medicine	0	39	0.782	1.579	0	34	0.666	1.349
Shopping	0	342	9.594	23.818	0	303	6.645	18.090
Sport	0	33	1.170	3.313	-	-	-	-
Transport (metro)	0	3	0.026	0.194	-	-	-	-
Transport (bus)#	0	56	2.416	5.484	0	8	0.172	0.632

#The number of day buses excludes night buses because of different operational periods.
